# Audio tagging of avian dawn chorus recordings in California, Oregon and Washington

**DOI:** 10.3897/BDJ.12.e118315

**Published:** 2024-04-29

**Authors:** Matthew James Weldy, Tom Denton, Abram B. Fleishman, Jaclyn Tolchin, Matthew McKown, Robert S. Spaan, Zachary J. Ruff, Julianna M. A. Jenkins, Matthew G. Betts, Damon B. Lesmeister

**Affiliations:** 1 Department of Forest Ecosystems and Society, Oregon State University, Corvallis, Oregon, United States of America Department of Forest Ecosystems and Society, Oregon State University Corvallis, Oregon United States of America; 2 Pacific Northwest Research Station, USDA Forest Service, Corvallis, Oregon, United States of America Pacific Northwest Research Station, USDA Forest Service Corvallis, Oregon United States of America; 3 Google DeepMind, California, United States of America Google DeepMind California United States of America; 4 Conservation Metrics Inc., Santa Cruz, California, United States of America Conservation Metrics Inc. Santa Cruz, California United States of America; 5 Department of Fisheries, Wildlife, and Conservation Sciences, Corvallis, Oregon, United States of America Department of Fisheries, Wildlife, and Conservation Sciences Corvallis, Oregon United States of America

**Keywords:** annotated soundscapes, forest ecology, amphibian, mammal, bird, vocalisation

## Abstract

**Background:**

Declines in biodiversity and ecosystem health due to climate change are raising urgent concerns. In response, large-scale multispecies monitoring programmes are being implemented that increasingly adopt sensor-based approaches such as acoustic recording. These approaches rely heavily on ecological data science. However, developing reliable algorithms for processing sensor-based data relies heavily on labelled datasets of sufficient quality and quantity. We present a dataset of 1,575 dawn chorus soundscape recordings, 141 being fully annotated (n = 32,994 annotations) with avian, mammalian and amphibian vocalisations. The remaining recordings were included to facilitate novel research applications. These recordings are paired with 48 site-level climatic, forest structure and topographic covariates. This dataset provides a valuable resource to researchers developing acoustic classification algorithms or studying biodiversity and wildlife behaviour and its relationship to environmental gradients. The dawn chorus recordings were collected as part of a long-term Northern Spotted Owl monitoring program; this demonstrates the complementary value of harnessing existing monitoring efforts to strengthen biodiversity sampling.

**New information:**

This dataset of dawn chorus soundscape recordings is one of the few open-access acoustic datasets annotated with non-biotic and both interspecific (across species) and intraspecific (within species) bird, mammal and amphibian sonotypes and the first that is paired with climatic, forest structure and topographical covariates extracted at recorder locations. This makes it a valuable resource for researchers studying the dawn chorus and its relationship to the environment.

## Introduction

Scientists worldwide are documenting unprecedented and potentially accelerating declines in ecosystem health and biodiversity (Butchart et al. 2010, Ceballos et al. 2020, Cowie et al. 2022), aggravated by climate change and other anthropogenic stressors (e.g. habitat loss and fragmentation). These declines have raised concerns about the loss of ecosystem integrity and the erosion of ecosystem services (Cardinale et al. 2012, Díaz et al. 2019), which has prompted increased demands for large-scale multispecies monitoring (Oliver et al. 2021). However, conventional monitoring methods are time-consuming, expensive and difficult to scale, which limits their spatial coverage, temporal resolution and species diversity.

To overcome these limitations, monitoring and research programmes increasingly adopt innovative protocols that leverage high-throughput sensor technologies like autonomous recording units and motion-activated cameras (Sugai et al. 2019, Tosa et al. 2021, Tuia et al. 2022). These sensor-based monitoring approaches offer several advantages over conventional methods (Shonfield and Bayne 2017, Gibb et al. 2018). These include lower per-unit sampling costs, archivable raw data, reduced invasiveness, the potential for expanded species coverage when paired with machine-learning classification algorithms and increased spatiotemporal sampling scales (provided that programmes choose to leverage the reduced per-unit cost to expand sampling scales). However, sensor-based data types are often unfamiliar to ecologists and may present new analytical and computational challenges that require interdisciplinary collaboration to develop and apply computational perception models (i.e. computer vision and computer hearing; Thessen 2016, Priyadarshani et al. 2018, Carey et al. 2019) and address changes in dataset size (Farley et al. 2018, Wall et al. 2021).

The quality of labelled datasets is essential for the successful training, evaluation and generalisation of perception models (Song et al. 2022, Mots'oehli and Baek 2023). The performance of a model improves with higher-quality data labels, regardless of the model architecture (Li et al. 2022). However, data preparation can be difficult and expensive (Stonebraker and Rezig 2019, Whang and Lee 2020), especially for ecological datasets that require specialised skills to identify specific sounds or image features (Thessen 2016). Labelled ecological datasets are increasingly being made available (Mumm and Knörnschild 2014, Fukushima et al. 2015, Goëau et al. 2016, Prat et al. 2017, Stowell et al. 2018, LeBien et al. 2020); however, only a few acoustic datasets focus on within-species sonotypes (Vidaña-Vila et al. 2017, Morfi et al. 2019) or specifically target the avian dawn chorus (Hopping et al. 2022). The avian dawn chorus remains challenging for computer hearing due to its complexity of calls, polyphonic character and potential for geographic variation (Duan et al. 2013, Stowell 2022). More dawn chorus-focused and vocalisation-specific acoustic datasets could help advance our understanding of an ecologically important period for the study of avian biodiversity (Bibby et al. 2000) and behaviour (McNamara et al. 1987, Staicer et al. 1996, Zhang et al. 2015, Teixeira et al. 2019).

The Northwest Forest Plan (hereafter NWFP; U.S. Department of Agriculture Forest Service and U.S. Department of the Interior Bureau of Land Management 1994), adopted in 1994, marked a refocus of federal land management policies towards a more balanced approach, prioritising the protection and recovery of habitat for imperilled old-forest species and overall biodiversity (U.S. Department of Agriculture Forest Service and U.S. Department of the Interior Bureau Land Management 1994). Under the NWFP Effectiveness Monitoring Program, long-term monitoring of federally threatened Northern Spotted Owl *Strixoccidentaliscaurina* (Merriam, 1898); hereafter spotted owl; (U.S. Fish and Wildlife Service 2020) populations is required. The monitoring programme consisted of two phases (Lint et al. 1999). The first phase focused on estimating vital rates and demographic performance using mark-resight data of spotted owls from historical territories in eight study areas (Franklin et al. 2021). The second phase used passive acoustic monitoring to collect data to estimate spotted owl occupancy and habitat associations across its range (Lesmeister et al. 2021, Lesmeister and Jenkins 2022). The transition to the second monitoring phase marks a potential watershed moment for conserving and managing forested lands in the Pacific Northwest. Spotted owl conservation and management objectives continue to be met (Duchac et al. 2020, Appel et al. 2023, Weldy et al. 2023), while simultaneously providing valuable multispecies acoustic monitoring data for other objectives, such as monitoring other old-forest-associated species identified in the NWFP, supporting other state and national conservation directives (such as the National Forest Management Act; 94th Congress 2nd Session (1976)), complementing strategic conservation planning efforts (Law et al. 2021) and contributing to global biodiversity conservation and monitoring efforts (IPBES 2018, Conference of the Parties to the Convention on Biological Diversity 2022).

In this context, we present annotated passive acoustic monitoring data collected during 2022 to support long-term monitoring of federally threatened spotted owl populations under the NWFP Effective Monitoring Program. The data were collected with Wildlife Acoustics Song Meter SM4 autonomous recording units during the hour following sunrise at 525 sites in California, Oregon and Washington, USA (Wildlife Acoustics, Maynard, MA). Additionally, we obscured exact sampling locations to protect sensitive species, but we provided 48 forest-structure-related environmental covariates extracted at each recorder location. This dataset provides value for researchers involved in developing or evaluating acoustic classification algorithms and for those interested in exploring the spatial variation in species-specific vocalisation phonology or the relationships amongst occurrence, vocalization behaviour and environmental characteristics, and contributes to making ecological research more transparent and reproducible (Poisot et al. 2013, Kenall et al. 2014, Baker and Vincent 2019).

## Project description

### Design description

We focused on the avian dawn chorus, including both migratory and resident species. The dawn chorus, characterized by the singing of numerous birds during the early morning hours, is an ecologically important period for studying avian behaviour (McNamara et al. 1987, Staicer et al. 1996, Zhang et al. 2015) and monitoring avian biodiversity (Bibby et al. 2000). By incorporating the study of the dawn chorus into the existing monitoring efforts, we aim to enrich our understanding of avian populations and their interactions within the Pacific-Northwest, which, in turn, can increase understanding of how individual species and biodiversity are influenced by anthropogenic and climatic change.

## Sampling methods

### Study extent

**Figure 1.**
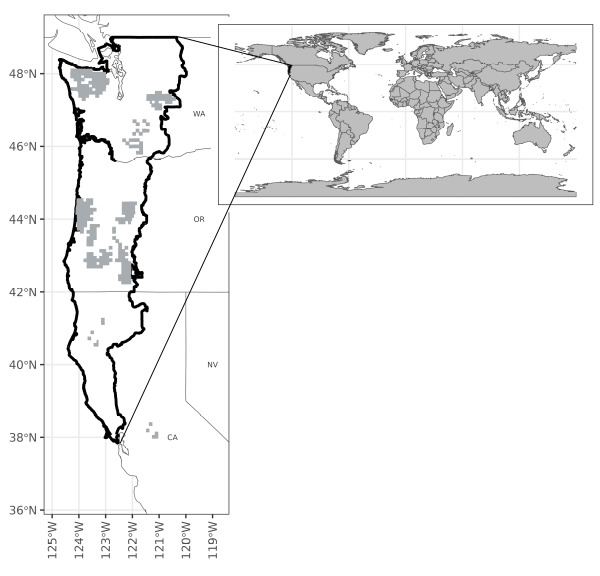
Map of acoustic recording locations (grey squares) in northern California, Oregon and Washington, USA. To protect sensitive species that might occur at our sampling locations, we obscured specific recording locations to the resolution of the overlapping Townships and Ranges.

In 2022, following the protocol outlined in Lesmeister et al. (2021), we collected acoustic recordings of 643 hexagons, each of which was 5 km^2^, that were randomly selected from a larger tessellation of hexagons covering the entire spotted owl range (northern California, Oregon and Washington; Fig. 1). We limited the set of available hexagons to those encompassing ≥ 50% forest-capable lands (defined as forested lands or lands capable of developing closed-canopy forests) and be under ≥ 25% federal ownership (Davis et al. 2011).

### Sampling description

Four Song Meter 4 (SM4) autonomous recording units were deployed in a standardised arrangement in each hexagon. The recorders were positioned at least 500 m apart at a minimum distance of 200 m from the hexagon boundary. The SM4 devices were mounted on to small trees (15–20 cm in diameter at breast height) approximately 1.5 m above the ground on mid-to-upper slopes and ≥ 50 m from roads, trails and streams. The SM4 devices have two built-in omnidirectional microphones with a signal-to-noise ratio of 80 dB, typical at 1 kHz and a recording bandwidth of 0.02–48 kHz. The recording rate was set to 32 kHz at 16-bit resolution and the data were saved in uncompressed WAV format.

Recordings were collected for six weeks, from March to August. Each device recorded approximately 11 hours of audio daily. The daily recording schedule comprised a 4-hour window starting two hours before sunrise and ending two hours after sunrise, another 4-hour window starting one hour before sunset and ending three hours after sunset and 10-minute recordings at the start of every hour outside the two longer recording blocks.

### Step description

**Dawn chorus dataset preparation**

We limited the available set of recordings to only those occurring from May–August to ensure we included migratory species (Robinson et al. 2019). This selection criteria resulted in a reduced pool of 525 hexagons. Three audio recordings from the first hour after sunrise were randomly selected from this subset and a 5-minute window was extracted from each recording using a randomised start time. The three recordings from each site are referred to as replicates. The resulting dataset comprised 1,575 x 5-minute recordings. We randomly selected 141 recordings from the dataset for full annotation (hereafter dawn chorus dataset; see dataset annotated_recordings.zip). Additional recordings (see dataset: additional_recordings_part_{1-11}.zip) were provided, either unlabelled or partially labelled, to facilitate novel research applications and methodological evaluations.

**Annotation methods**

Prior to labelling, we developed a sonotype library, which describes the acoustic properties of 260 sound types and describes a standardised label structure (see dataset: metadata.tsv). Sonotype descriptions and categories were developed by acoustic and visual inspection of examples and supplemented by descriptions provided by Pieplow (2019). We provide two label sets: the first provides species-level identifications, based on the 2021 eBird codes according to Clement’s taxonomy (Clements et al. 2022). The second describes different vocalisations within species by concatenating the 2021 eBird code for the species with codes that incremented depending on the species repertoire (i.e. ‘call_1,’ ‘song_1,’ ‘drum_1’). For example, ‘herthr_song_1’ is the label for Hermit Thrush *Catharusguttatus* (Pallus, 1811) song_1. Amphibian and mammalian common species names were adapted following the structure of the 2021 eBird codes.

Two trained annotators labelled each 2-second window with labels from the set of potential sound types. Unknown signals were labelled ‘unknown,’ and clips with no biotic signals (or noise classes of interest documented in metadata.tsv) were labelled ‘empty.’ Windows were labelled ‘complete’ and considered fully annotated when every signal was assigned an annotation. Files were deemed fully annotated when every 2-second window of the 5-minute recording was assigned the ‘complete’ label. The label 'impossible' was utilised for instances where a biological sound was present in the window, but could not be confidently identified, often due to faintness or being obscured by rain. Additionally, eight aggregated biotic sounds were not separated due to uncertainty in assigning a label confidently.

We used two annotation methods: linear annotation and model-assisted annotation. The linear annotation method consisted of fully annotating dawn chorus recordings in sequence. The model-assisted method used BirdNET version 2.2 (Kahl et al. 2021) using default settings with longitude and latitude set to -1 and a proprietary multi-label convolutional neural network classification model developed by Conservation Metrics, Inc., pre-trained avian classification models, to group audio windows with high-confidence predictions for common species. The grouped audio windows were manually reviewed so clips with common sound types could be reviewed concurrently. We did not record which model was used to search specific signals, but grouped by several expected species from each model and the "empty" category in the proprietary model. The model-assisted clips were then fully reviewed and labelled using the linear method and vocalisations for other species were added to the clip annotations.

**Environmental characteristics**

We included 48 variables, including three climatic variables, 38 forest structure variables, five topographic variables and two masked spatial variables. We downloaded estimates of annual precipitation (mm), minimum temperature (°C) and maximum temperature (°C) averaged from 1970–2000 at a 1 km^2^ resolution from WorldClim version 2.1 (Fick and Hijmans 2017). We downloaded estimates of forest structure characteristics from LEMMA (Landscape Ecology Modelling Mapping and Analysis Team (LEMMA) 2020). LEMMA forest structure estimates are derived using gradient nearest-neighbour imputation methods, based on regional inventory plots (Ohmann and Gregory 2002). For topographic variables, we downloaded digital elevation models (DEM) at 10 m^2^ resolution from Earth Explorer. Using a mosaicked DEM, we estimated slope, topographic position index (TPI), vector ruggedness measure (VRM) and northness at 10 m^2^ resolution. We calculated TPI, scaled from −39.1 to 43.8, as the mean difference of the central points to the focal squares of the surrounding 5 × 5 grid cells. Thus, low and high values represent lower and higher slopes, respectively (Wiess 1999). We then estimated VRM, which integrates the variation in slope and aspect, using the methods described in Sappington et al. (2010). VRM provides a better measure of terrain variability than slope and elevation (Sappington et al. 2010). As aspect is a circular variable, we transformed it into "northness" such that northness = cosine(aspect), which is scaled so that southern exposed lands have values close to −1 and northern exposed lands have values close to 1 (Guisan et al. 1999, Lassueur et al. 2006). All spatial data transformations and extractions were completed using the terra (1.7–39) and sf (1.0–14) packages for R version 4.1.2 (Pebesma 2018, R Core Team 2021, Hijmans 2023, Pebesma and Bivand 2023).

**Analytical Methods**

We performed two sets of analyses. The first set of analyses used t-distributed stochastic neighbour (van der Maaten and Hinton 2008, Hinton and Roweis 2022) to visualise BirdNET version 2.2 embeddings (Kahl et al. 2021) for three aggregated biophonic sound groups. The first visualisation characterised the Parulidae complex (ebird code: paruli) and the *Setophaga* complex (ebird code: setoph). The Parulidae complex label was assigned to acoustic clips to which we could not confidently assign labels, but included sounds from macwar, naswar, wlswar and yerwar. Similarly, the *Setophaga* label was assigned to acoustic clips to which we could not confidently assign labels, but included sounds from btywar, herwar and towwar. The second visualisation characterised the unknown avian chip call class, including examples of avian chip calls to which we could not confidently assign labels. We extracted feature embeddings for each clip in both visualisations using BirdNET version 2.2. The 320-dimensional feature embeddings were then mapped to two dimensions using t-distributed stochastic neighbour embedding using a principal components initialisation and fit with 5,000 iterations and a perplexity value of 20. The second analysis estimated the difference in the Gaussian kernel density of species-specific occurrence across three gradients of environmental characteristics. The base rate kernel density for the sampling occurrence was subtracted from the species-specific occurrence kernel density to evaluate if species occurred more frequently at specific values of environmental characteristics relative to the representation of the environmental characteristic across the sample locations. Values greater than one indicate a higher occurrence relative to the base rate and values less than one indicate a lower occurrence relative to the base rate.

## Geographic coverage

### Description

We used acoustic recordings collected from federally managed lands in California, Oregon and Washington, focusing on forest-capable areas (Fig. 1). The recording locations were randomly selected within a defined bounding box, spanning latitudes from 37°43'48''N to 49°01'48''N and longitudes from 125°00'00''W to 120°30'00''W. Elevations at the recording locations ranged 52 to 2,252 m.

We took measures to protect sensitive species that might occur at our sampling locations. As a result, we obscured specific recording locations to the resolution of the overlapping Townships and Ranges, which are approximately 1 mi^2^ (2.58 km^2^) grid cells used by the U.S. Public Land Survey System. We also provide detailed environmental characteristics extracted from the actual recorder locations. This approach ensures data privacy, while allowing us to furnish essential information for our study.

### Coordinates

37°43'48''N and 49°01'48''N Latitude; 125°00'00''W and 120°30' 00''W Longitude.

## Taxonomic coverage

### Description

We identified 116 sound types during the annotation of these recordings (Table 1). The annotations include sonotypes from 58 avian species, two mammalian species, one amphibian species, eight aggregated biophonic sounds, one geophonic sound type and six anthrophonic sound types (Table 2). The eight aggregated biotic sounds included the labels “chipmu”, “drum”, “fly”, “paruli”, “setoph”, “tree” and “unk”. The "paruli" and "setoph" labels consisted of ambiguous sounds similar to those of other unambiguous labels. The other aggregated biotic labels include sounds made by multiple species; however, because the sounds described by each label type were similar, we could not assign labels at a finer taxonomic resolution.

## Temporal coverage

**Data range:** 2022-5-01 – 2022-9-25.

### Notes

The audio clips comprising this dataset were recorded during the initial hour following sunrise, spanning the time frame from 01-05-2022 to 25-09-2022. However, due to variations in the spatial distribution of our recording units and the effects of our filtering criteria, recordings from May are relatively over-represented and recordings from California only occurred during May and June (Table 1).

## Usage licence

### Usage licence

Other

### IP rights notes

Creative Commons Attribution (CC-BY) 4.0 License

## Data resources

### Data package title

Audio tagging of avian dawn chorus recordings in California, Oregon and Washington

### Resource link

DOI: https://zenodo.org/doi/10.5281/zenodo.8047849

### Number of data sets

7

### Data set 1.

#### Data set name

Acoustic files

#### Data format

tsv

#### Download URL

https://zenodo.org/records/10895837/files/acoustic_files.tsv?download=1

#### Description

This dataset describes the acoustic recordings included in this dataset. The acoustic recordings described in the dataset are available through an online data repository DOI: https://zenodo.org/doi/10.5281/zenodo.8047849.

**Data set 1.** 

Column label	Column description
site	Site name.
replicate	An ordinal label indicating the random draw label: ‘A’, ‘B’, or ‘C’.
recording_date	Recording date and time formatted as “Year-Month-Day Hour:Minute:Second”.
annotated	Categorical assignment describing whether a recording was completely annotated: ‘complete,’ ‘partial,’ or ‘not annotated’.
file	Wav file name.
zip_file	The zip file location of the file.

### Data set 2.

#### Data set name

Acoustic annotations

#### Data format

tsv

#### Download URL

https://zenodo.org/records/10895837/files/acoustic_annotations.tsv?download=1

#### Description

This dataset lists all annotations from the fully annotated recordings.

**Data set 2.** 

Column label	Column description
file	Wav file name.
start	Start time of the 2-second clip in seconds.
end	End time of the 2-second clip in seconds.
eBird_2021	2021 species identification eBird code.
label	Sonotype label concatenates the 2021 eBird taxonomy code and the sound type label.

### Data set 3.

#### Data set name

Partial annotations

#### Data format

tsv

#### Download URL

https://zenodo.org/records/10895837/files/partial_annotations.tsv?download=1

#### Description

This dataset lists all annotations from the partially annotated recordings.

**Data set 3.** 

Column label	Column description
file	Wav file name.
start	Start time of the 2-second clip in seconds.
end	End time of the 2-second clip in seconds.
clip_complete	Binary indicator for whether the clip was completely labelled.
eBird_2021	2021 species identification eBird code.
label	Sonotype label comprising a concatenation of the 2021 eBird taxonomy code and the sound type label.

### Data set 4.

#### Data set name

Annotation metadata

#### Data format

tsv

#### Download URL

https://zenodo.org/records/10895837/files/annotation_metadata.tsv?download=1

#### Description

This dataset describes the focal acoustic sounds included in the recording annotations.

**Data set 4.** 

Column label	Column description
label	Sonotype label comprising a concatenation of the 2021 eBird taxonomy code and the sound type label.
eBird_2021	2021 eBird taxonomy species_code.
sound	Sound type label.
common_name	The common name of the sound source. For avian species, the scientific name follows Clement’s taxonomy outlined in the 2021 eBird taxonomy.
scientific_name	The scientific name of the biotic sound source. For avian species, the scientific name follows Clement’s taxonomy outlined in the 2021 eBird taxonomy.
taxonomic_authority	Primary taxonomic authority.
description	Biological and phonetic description of the target sound.
n_files	Total number of audio files containing at least one of the target labels.
n_annotations	Total number of label-specific annotations in the fully annotated data.

### Data set 5.

#### Data set name

Environmental characteristics

#### Data format

tsv

#### Download URL

https://zenodo.org/records/10895837/files/environmental_characteristics.tsv?download=1

#### Description

This dataset lists the environmental characteristics at each recording station. Units of measurements for appropriate covariates are in parentheses.

**Data set 5.** 

Column label	Column description
site	Site name.
replicate	An ordinal label indicating whether the row describes a random sample ‘A’, ‘B’ or ‘C’.
state	State location of survey site.
township_range	Township and range identifier of the survey site. The township was data obtained from three sources: CA, OR, WA.
age_dom_2017	Basal area weighted stand age, based on dominant and codominant trees (years).
ba_ge_3_2017	Basal area of live trees >= 2.5 cm dbh (m^2^/ha).
bac_ge_3_2017	Basal area of live conifers >= 2.5 cm dbh (m^2^/ha).
bah_ge_3_2017	Basal area of live hardwoods >= 2.5cm dbh (m^2^/ha).
bph_ge_3_crm_2017	Component Ratio Method biomass of all live trees >= 2.5 cm (kg/ha).
bphc_ge_3_crm_2017	Component Ratio Method biomass of all live conifers >= 2.5 cm (kg/ha).
bphh_ge_3_crm_2017	Component Ratio Method biomass of all live hardwoods >= 2.5 cm (kg/ha).
cancov_2017	Canopy cover of all live trees (percent).
cancov_con_2017	Canopy cover of all conifers (percent).
cancov_hdw_2017	Canopy cover of all hardwoods (percent).
cancov_layers_2017	Number of tree canopy layers present (number of layers).
conplba_2017	Conifer tree species with the plurality of basal area (raster to alphanumeric look-up table available at source).
covcl_2017	Cover class based on cancov (raster to alphanumeric look-up table available at source).
ddi_2017	Diameter diversity index
fortypba_2017	Forest type, which describes the dominant tree species of current vegetation (raster to alphanumeric look-up table available at source).
hdwplba_2017	Hardwood tree species with the plurality of basal area (raster to alphanumeric look-up table available at source).
mndbhba_2017	Basal-area weighted mean diameter of all live trees (cm).
mndbhba_con_2017	Basal-area weighted mean diameter of all live conifers (cm).
mndbhba_hdw_2017	Basal-area weighted mean diameter of all live hardwoods (cm).
qmd_dom_2017	The quadratic mean diameter of all dominant and codominant trees (cm).
qmd_ht25_2017	The quadratic mean diameter in inches of trees whose heights are in the top 25% of all tree heights (cm).
qmdc_dom_2017	The quadratic mean diameter of all dominant and codominant conifers (cm).
qmdh_dom_2017	The quadratic mean diameter of all dominant and codominant hardwoods (cm).
sbph_ge_25_2017	Biomass of snags >= 25 cm dbh and >= 2m tall (lb).
sdi_reineke_2017	Reineke's stand density index.
sizecl_2017	Size class, based on qmd_dom and cancov (raster to alphanumeric look-up table available at source).
stndhgt_2017	Stand height, computed as the average height of all dominant and codominant trees (m).
stph_ge_25_2017	Density of snags >= 25 cm dbh and >= 2 m tall (trees/ha).
struccond_2017	Structural condition (raster to alphanumeric look-up table available at source).
svph_ge_25_2017	Volume of snags >= 25 cm dbh and >= 2 m tall (m^3^/ha).
tph_ge_3_2017	The density of live trees >= 2.5 cm dbh (trees/ha).
tphc_ge_3_2017	The density of live conifers >= 2.5 cm dbh (trees/ha).
tphh_ge_3_2017	The density of live hardwoods >= 2.5 cm dbh (trees/ha).
treeplba_2017	Tree species with the plurality of basal area (raster to alphanumeric look-up table available at source).
vegclass_2017	Vegetation class based on cancov, bah_prop, qmd_dom (raster to alphanumeric look-up table available at source).
vph_ge_3_2017	The volume of live trees >= 2.5 cm dbh (m^3^/ha).
vphc_ge_3_2017	The volume of live conifers >= 2.5 cm dbh (m^3^/ha).
vphh_ge_3_2017	The volume of live hardwoods >= 2.5 cm dbh (m^3^/ha).
dem_30m	Digital elevation model at 30 m² resolution (m).
northness_30m	A cosine transformation of aspect to demonstrate the orientation of a land relative to a north-facing land derived from dem_30m.
slope_30m	Estimate of land slope at 30 m² resolution derived from dem_30m.
tpi5x5_30m	Mean difference of the central point to a focal square of the surrounding 5 × 5 grid cells derived from dem_30m.
vrm_30m	Variation in slope and aspect derived from dem_30m.
an_precip_1km	Average precipitation at a 1 km^2^ resolution averaged from 1970-2000 (mm).
minT_1km	Average minimum temperature at a 1 km^2^ resolution averaged from 1970-2000 (degrees Celcius).
maxT_1km	Average maximum temperature at a 1 km^2^ resolution averaged from 1970-2000 (degrees Celcius).

### Data set 6.

#### Data set name

Environmental characteristics metadata

#### Data format

tsv

#### Download URL

https://zenodo.org/records/10895837/files/environmental_characteristics_metadata.tsv?download=1

#### Description

This dataset describes the environmental characteristics included in environmental_characteristics.

**Data set 6.** 

Column label	Column description
covariate	Covariate name.
type	Value type of variable.
range	The range of values extracted across our survey sites. The values in this cell represent the value minimum to the value maximum.
unit	A description of the variable units of measurement.
description	A description of the variable, including a brief discussion of the methods used to create the variable.
source	Variable source.

### Data set 7.

#### Data set name

Annotator identification and annotation method

#### Data format

tsv

#### Download URL

https://zenodo.org/records/10895837/files/annotator_method.tsv?download=1

#### Description

This dataset describes the annotator identification and annotation method for each 2-second window.

**Data set 7.** 

Column label	Column description
file	Wav file name.
start	Start time of the 2-second clip in seconds.
end	End time of the 2-second clip in seconds.
method	The annotation method used to label the 2-second clip. This label is only available for a subset of clips used to estimate annotation speed.
annotator	The annotator identifier for the 2-second clip.

## Additional information

### Acoustic recordings

The fully annotated acoustic recordings are available for download in a zip file of uncompressed wav format files.

- annotated_recordings.zip (141 WAV files, 1.8 GB)

The partial and unannotated recordings are available in 11 zip files of uncompressed wav format files.

- additional_recordings_part_1.zip (132 WAV files, 1.6 GB)
- additional_recordings_part_2.zip (139 WAV files, 1.7 GB)
- additional_recordings_part_3.zip (137 WAV files, 1.7 GB)
- additional_recordings_part_4.zip (139 WAV files, 1.8 GB)
- additional_recordings_part_5.zip (139 WAV files, 1.8 GB)
- additional_recordings_part_6.zip (140 WAV files, 1.9 GB)
- additional_recordings_part_7.zip (139 WAV files, 1.8 GB)
- additional_recordings_part_8.zip (131 WAV files, 1.7 GB)
- additional_recordings_part_9.zip (135 WAV files, 1.8 GB)
- additional_recordings_part_10.zip (133 WAV files, 1.7 GB)
- additional_recordings_part_11.zip (70 WAV files, 900.4 MB)

### Data dictionaries

Descriptive data dictionaries are available for download as a pdf file.

- data_dictionaries.pdf (63 kB)

### Results and Discussion

**Figure 2.**
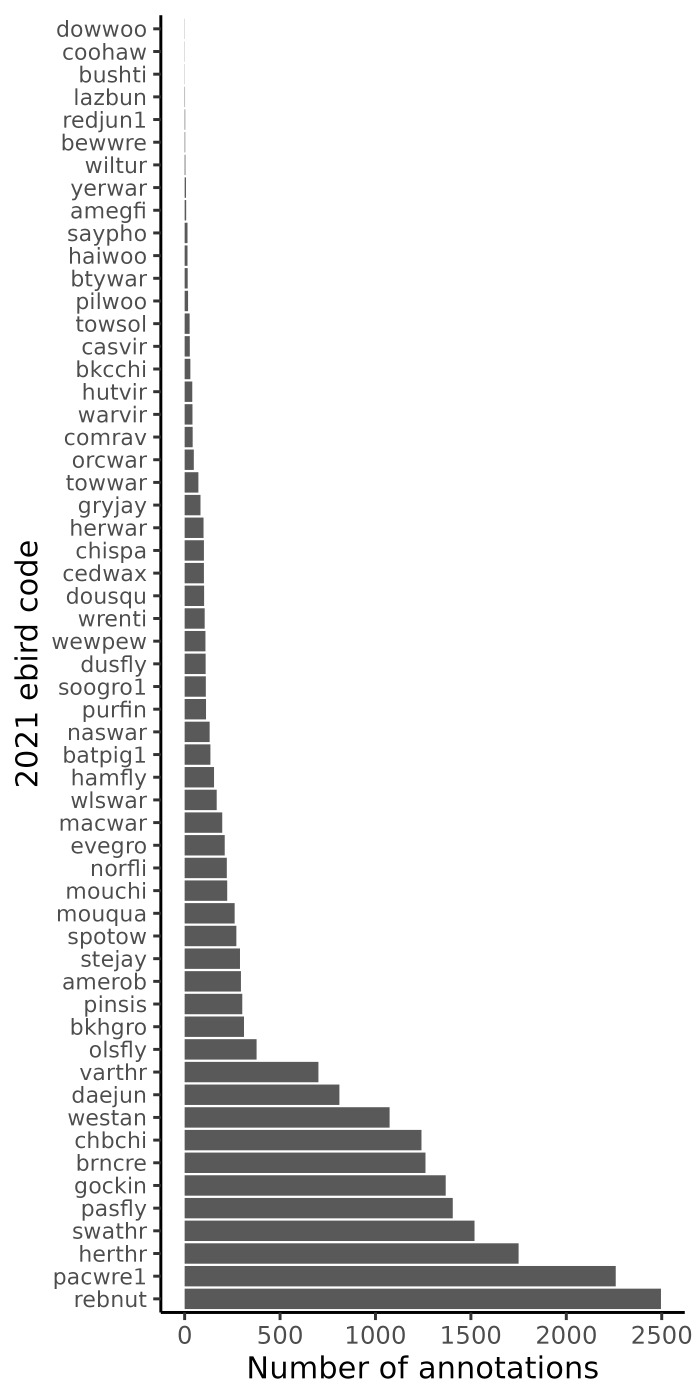
This vertical barplot visualises the frequency of annotations for the most prevalent species within the annotated dataset. The y-axis lists species by their 2021 eBird codes, ordered from most to least frequent (see Table 1 for common names). The x-axis displays the cumulative annotation count for each species. More prevalent species occur towards the bottom and have higher annotation counts. The plot reveals that a few common species dominate annotations, while many are annotated infrequently.

We fully annotated 11.75 hours of audio with 32,994 labels for 115 sonotypes. An additional 216 files were partially annotated with 5,278 labels for 53 sonotypes. We also provide 20,737 auditing labels indicating clip-level completion status. The most frequently annotated species were Red-breasted Nuthatch *Sittacanadensis* (Linnaeus, 1766; eBird code: rebnut; n annotations = 2,496), Pacific Wren *Troglodytespacificus* (Baird, 1864; eBird code: pacwre1; n annotations = 2,259), Hermit Thrush (eBird code: herthr; n annotations = 1,750), Swainson’s Thrush *Catharusustulatus* (Nuttall, 1840; eBird code: swathr; n annotations = 1,519), Pacific-slope Flycatcher *Empidonaxdifficilis* (Baird, 1858; eBird code: pasfly; n annotations = 1,405) and Golden-crowned Kinglet *Regulussatrapa* (Lichtenstein, 1823; eBird code: gockin; n annotations = 1,368; Fig. 2). There were 25 classes with fewer than 10 annotations (Fig. 2).

We annotated an average of 695 windows per hour (σ = 363). However, the annotation rate varied between annotators and methods. The model-assisted method appeared to increase the rate for both annotators relative to the linear method (Table 3); however, estimates are from just two annotators across 84 x 5-minute clips. As the model-produced labels are imperfect, all segments were reviewed by a human annotator to confirm or deny the candidate labels. Without review, biases in the trained model could be passed on to the new model as a type of confirmation bias (Ouali et al. 2020) and new biases may be introduced through distribution shifts (Gibb et al. 2023). However, collecting similar windows simplified the annotation process by narrowing the label search space.

**Figure 3.**
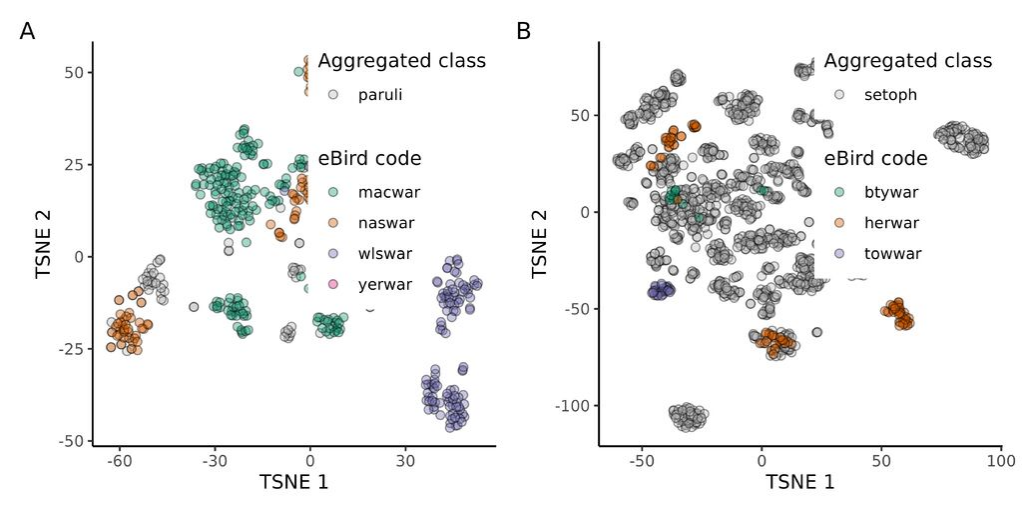
Two-dimensional t-SNE (t-distributed stochastic neighbour embedding) plots of the BirdNET embeddings for two aggregated biotic classes and unambiguous examples from individual species included in the aggregated classes. Each data point on the plot corresponds to an individual 2-second audio clip. Panel A plots the t-SNE embedding for the paruli aggregated class, which includes MacGillivray's Warbler *Geothlypistolmiei* (Townsend, 1839; eBird code: macwar), Nashville Warbler *Leiothlypisruficapilla* (Wilson, 1811; eBird code: naswar), Yellow-rumped Warbler *Setophagacoronata* (Linnaeus, 1766; eBird code: yerwar) and Wilson's Warbler *Cardellinapusilla* (Wilson, 1811; eBird code: wlswar). Panel B plots the t-SNE embedding for the *Setophaga* aggregated class, which includes Hermit Warbler *Setophagaoccidentalis* (Townsend, 1837; eBird code: herwar), Townsend's Warbler *Setophagatownsendi* (Townsend, 1837; eBird code: towwar) and Black-throated Gray Warbler *Setophaganigrescens* (Townsend, 1837; eBird code: btywar). This visualisation compares the aggregated classes to known examples from key species, evaluating the overlap of individual species embeddings relative to their assigned aggregated class.

**Figure 4.**
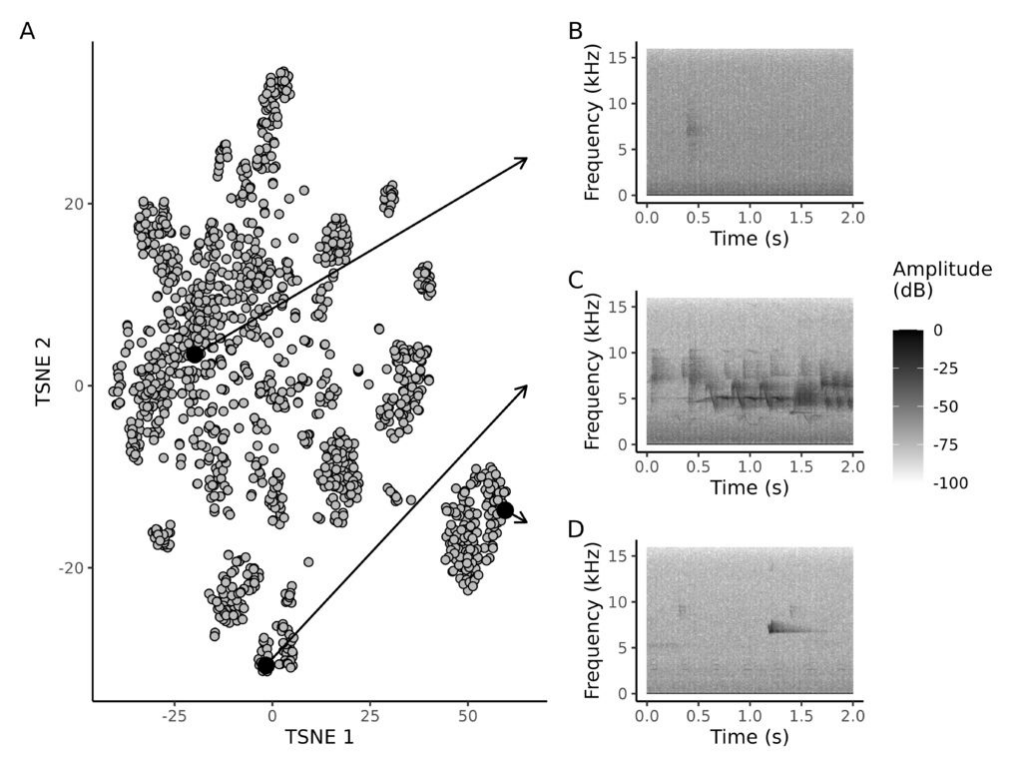
Two-dimensional t-SNE (t-distributed stochastic neighbour embedding) plots of the birdnet embeddings for the aggregated biotic unknown avian chip vocalisation class (eBird code: unk). Each data point on the plot corresponds to an individual 2-second audio clip. Panels B, C and D provide detailed spectrograms for selected audio clips marked by opaque black points on the t-SNE plot. Panel B exemplifies a typical audio clip near the centre of the primary unknown chip cluster within the t-SNE plot. Many audio clips in this cluster contain only an avian chip vocalisation. Panel C features the spectrogram of an audio clip from the most negative sub-cluster along the t-SNE axis 2. Audio clips within this sub-cluster primarily contain vocalisations from the aggregated *Setophaga* class. Panel D displays the spectrogram of an audio clip from the most positive cluster along t-SNE axis 1. Audio clips within this cluster predominantly consist of Red-breasted Nuthatch vocalisations.

Two of the aggregated biophonic sound groups (Parulidae complex: paruli, *Setophaga* complex: setoph) consisted of groups of similar sound types (eBird codes for sound classes included in the Parulidae complex: macwar, naswar, wlswar, yerwar; eBird codes for sound classes included in the *Setophaga* complex: btywar, herwar, towwar) that we were unable to assign to a species-level eBird code confidently. Another aggregated biophonic sound (Unknown chip: unk) consisted of unknown avian chip calls, which we were also unable to assign to a species-level eBird code confidently. We could not confidently differentiate six biotic sound groups. To gain insight into the acoustic structure of these groups, we used t-distributed stochastic neighbour embedding (t-SNE) of BirdNET embeddings (Hinton and Roweis 2022) to visualise the acoustic geometry. t-SNE is a dimensionality reduction technique that projects high-dimensional data into lower dimensions while attempting to preserve local distances between data points (van der Maaten and Hinton 2008). t-SNE visualisations can be challenging to interpret because global distances are not always preserved, but appropriate initialisation can improve global representations (Kobak and Linderman 2021). We found that both aggregated groups of warbler vocalisations overlapped with known examples of warbler vocalisations (Fig. 3). Notably, Wilson’s Warbler *Cardellinapusilla* (Wilson, 1811; eBird code: wlswar) songs displayed the greatest distinction within the paruli complex. Unknown avian chip vocalisations generally clustered together and outlying clusters were polyphonic with additional bird songs (Fig. 4). For example, one cluster contained *Setophaga* songs (Fig. 4, Panel C), while another contained Red-breasted Nuthatch songs (Fig. 4, Panel D).

**Figure 5.**
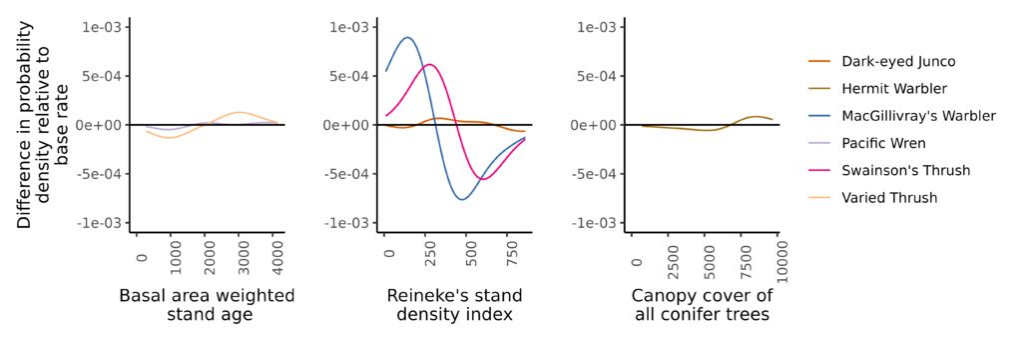
Kernel density plots of species occurrence across gradients of Basal area weighted stand age (bandwidth = 2000), Reineke’s stand density index (bandwidth = 500) and canopy cover of conifer trees (bandwidth = 5000). The species-specific probability densities are shown relative to the base rate of sampling occurrence across each environmental gradient. Specialist species with respect to an environmental gradient should show higher or lower probabilities relative to the sampling base rate within some range of the environmental gradient (i.e. > or < 0), whereas generalist species for a given environmental gradient should match the sampling base rate of occurrence (i.e. ~ 0).

In its essence, a labelled acoustic dataset is a presence-absence dataset. When we pair species-level labels with local environmental characteristics, we can explore the relative presence of species across environmental gradients. For example, Varied Thrush *Ixoreusnaevius* (Gmelin, 1789) and Pacific Wren prefer older forests, implying that their likelihood of occurrence within such habitats should be higher when compared to the baseline sampling rate of older forests and, for any given covariate, a species with a typical response pattern should closely align with that baseline sampling rate (Fig. 5; Hansen et al. (1995)). Furthermore, environmental covariates are commonly used as training features in ecological models, where covariation amongst response variables and environmental covariates is leveraged to distinguish and predict behavioural, occurrence and demographic patterns. For difficult to distinguish acoustic classes, incorporating environmental features with embeddings could improve differentiation by providing contextual information, as demonstrated in other fields (Tang et al. 2015, Liu et al. 2018, Aodha et al. 2019, Terry et al. 2020). For example, Jeantet and Dufourq (2023) incorporated geographic and temporal contextual information into convolutional neural network-based acoustic classifiers in a multibranch network structure and observed decreases in false-positive rates and significant improvements in detection rates for bird songs and Hainan gibbon *Nomascushainanus* (Thomas, 1892) calls in contextualised models relative to non-contextualised baseline models. In another example, Knight et al. (2020) incorporated signal energy into a post-processing predictive validation procedure, which could be extended to include environmental characteristics.

### Conclusion

Recent advances in computational algorithms have made passive acoustic monitoring more accessible (Kahl et al. 2021, Ghani et al. 2023. In part, these advances have been driven by increasing data availability. However, differentiating vocalisation types within species and detecting vocalisations during periods of high vocal activity, such as the dawn chorus, remain challenging (Joly et al. 2019). To address this, we present an acoustic dataset focusing on these two challenges. We annotated recordings from the avian dawn chorus period and paired them with environmental covariates at each recording location. The dataset includes labels for within-species vocalisation types and annotations beyond the avian community. We also provide additional unlabelled acoustic files that can be used in the development of novel machine-learning applications.

## Figures and Tables

**Table 1.** This dataset includes annotations for 116 sound categories, including 58 avian species, two mammalian species, one amphibian species, eight aggregated biophonic sounds, one geophonic sound type and six anthrophonic sound types. Each annotation is accompanied by its corresponding sound type, common name, scientific name and species code, following the 2021 eBird conventions for Clement's taxonomy. Astericks (*) indicate novel class labels following Clement’s naming conventions.

Type	Common name	Scientific name	eBird code
Aggregated Biophonic	Chipmunk	*Neotamias* spp.	chipmu*
	Drum	Picidae	drum*
	Fly	Insecta	fly*
	Parulidae complex	Parulidae	paruli*
	*Setophaga* complex	*Setophaga* spp.	setoph*
	Tree creak		tree*
	Unknown chip	Aves	unk*
	Wingbeat	Aves	wingbeat*
Amphibian	American Bullfrog	*Ranacatesbeiana* (Shaw, 1802)	amebul*
Anthrophony	Airplane		airplane*
	Chainsaw		chainsaw*
	Gunshot		gunshot*
	Sensor noise		sensor*
	Truck beep		truck*
	Vehicle		vehicle*
Bird	American Goldfinch	*Spinustristis* (Linnaeus, 1758)	amegfi
	American Robin	*Turdusmigratorius* (Linnaeus, 1766)	amerob
	Band-tailed Pigeon	*Patagioenasfasciata* (Say, 1822)	batpig1
	Bewick's Wren	*Thryomanesbewickii* (Audubon, 1827)	bewwre
	Black-capped Chickadee	*Poecileatricapillus* (Linnaeus, 1766)	bkcchi
	Black-headed Grosbeak	*Pheucticusmelanocephalus* (Swainson, 1827)	bkhgro
	Black-throated Gray Warbler	*Setophaganigrescens* (Townsend, 1837)	btywar
	Brown Creeper	*Certhiaamericana* (Bonaparte, 1838)	brncre
	Bushtit	*Psaltriparusminimus* (Townsend, 1837)	bushti
	Canada Jay	*Perisoreuscanadensis* (Linnaeus, 1766)	gryjay
	Cassin's Vireo	*Vireocassinii* (Xantus de Vesey, 1858)	casvir
	Cedar Waxwing	*Bombycillacedrorum* (Vieillot, 1808)	cedwax
	Chestnut-backed Chickadee	*Poecilerufescens* (Townsend, 1837)	chbchi
	Chipping Sparrow	*Spizellapasserina* (Bechstein, 1798)	chispa
	Common Raven	*Corvuscorax* (Linnaeus, 1758)	comrav
	Cooper's Hawk	*Accipitercooperii* (Bonaparte, 1828)	coohaw
	Dark-eyed Junco	*Juncohyemalis* (Linnaeus, 1758)	daejun
	Downy Woodpecker	*Dryobatespubescens* (Linnaeus, 1766)	dowwoo
	Dusky Flycatcher	*Empidonaxoberholseri* (Phillips, 1939)	dusfly
	Evening Grosbeak	*Hesperiphonavespertina* (Cooper, 1825)	evegro
	Golden-crowned Kinglet	*Regulussatrapa* (Lichtenstein, 1823)	gockin
	Hairy Woodpecker	*Leuconotopicusvillosus* (Linnaeus, 1766)	haiwoo
	Hammond's Flycatcher	*Empidonaxhammondii* (Xantus de Vesey, 1858)	hamfly
	Hermit Thrush	*Catharusguttatus* (Pallus, 1811)	herthr
	Hermit Warbler	*Setophagaoccidentalis* (Townsend, 1837)	herwar
	Hutton's Vireo	*Vireohuttoni* (Cassin, 1851)	hutvir
	Lazuli Bunting	*Passerinaamoena* (Say, 1822)	lazbun
	MacGillivray's Warbler	*Geothlypistolmiei* (Townsend, 1839)	macwar
	Mountain Chickadee	*Poecilegambeli* (Ridgway, 1886)	mouchi
	Mountain Quail	*Oreortyxpictus* (Douglas, 1829)	mouqua
	Nashville Warbler	*Leiothlypisruficapilla* (Wilson, 1811)	naswar
	Northern Flicker	*Colaptesauratus* (Linnaeus, 1758)	norfli
	Northern Pygmy-Owl	*Glaucidiumgnoma* (Wagler, 1832)	nopowl
	Olive-sided Flycatcher	*Contopuscooperi* (Nuttall, 1831)	olsfly
	Orange-crowned Warbler	*Leiothlypiscelata* (Say, 1822)	orcwar
	Pacific Wren	*Troglodytespacificus* (Baird, 1864)	pacwre1
	Pacific-slope Flycatcher	*Empidonaxdifficilis* (Baird, 1858)	pasfly
	Pileated Woodpecker	*Dryocopuspileatus* (Linnaeus, 1758)	pilwoo
	Pine Siskin	*Spinuspinus* (Wilson, 1810)	pinsis
	Purple Finch	*Haemorhouspurpureus* (Gmelin, 1789)	purfin
	Red-breasted Nuthatch	*Sittacanadensis* (Linnaeus, 1766)	rebnut
	Rooster (Red Junglefowl)	*Gallusgallus* (Linnaeus, 1758)	redjun1
	Rufous Hummingbird	*Selasphorusrufus* (Gmelin, 1788)	rufhum
	Say's Phoebe	*Sayornissaya* (Bonaparte, 1825)	saypho
	Sooty Grouse	*Dendragapusfuliginosus* (Ridgway, 1873)	soogro1
	Spotted Towhee	*Pipilomaculatus* (Swainson, 1827)	spotow
	Steller's Jay	*Cyanocittastelleri* (Gmelin, 1788)	stejay
	Swainson's Thrush	*Catharusustulatus* (Nuttall, 1840)	swathr
	Townsend's Solitaire	*Myadestestownsendi* (Audubon, 1838)	towsol
	Townsend's Warbler	*Setophagatownsendi* (Townsend, 1837)	towwar
	Varied Thrush	*Ixoreusnaevius* (Gmelin, 1789)	varthr
	Warbling Vireo	*Vireogilvus* (Vieillot, 1808)	warvir
	Western Tanager	*Pirangaludoviciana* (Wilson, 1811)	westan
	Western Wood-Pewee	*Contopussordidulus* (Sclater, 1859)	wewpew
	Wild Turkey	*Meleagrisgallopavo* (Linnaeus, 1758)	wiltur
	Wilson's Warbler	*Cardellinapusilla* (Wilson, 1811)	wlswar
	Wrentit	*Chamaeafasciata* (Gambel, 1845)	wrenti
	Yellow-rumped Warbler	*Setophagacoronata* (Linnaeus, 1766)	yerwar
Geophony	Rain		rain*
Mammal	Dog	*Canislupusfamiliaris* (Linnaeus, 1758)	dog*
	Douglas squirrel	*Tamiasciurusdouglasii* (Bachman, 1839)	dousqu*

**Table 2.** Number of acoustic recordings collected from May to August 2022.

State	May	June	July	August
California	19	8	0	0
Oregon	487	222	278	0
Washington	187	122	191	61

**Table 3.** Summary statistics for the annotation rate, measured in windows annotated per hour, from two annotators employing both linear and model-assisted annotation protocols. The summary includes the following metrics: mean, standard deviation (sd), minimum rate (min.) and maximum rate (max.).

Annotation method	Annotator	mean	sd	min.	max.
linear	1	651.6	274.5	333.3	1125.0
	2	580.3	605.3	160.7	2250.0
model-assist	1	883.8	292.2	303.2	1582.4
	2	496.3	227.0	201.4	1170.7
